# Effects of Xin-Ji-Er-Kang on Anticardiovascular Remodeling in* L*-NAME Induced Hypertensive Mice and Its Potential Mechanisms

**DOI:** 10.1155/2018/8067361

**Published:** 2018-02-28

**Authors:** Li Wang, Guo-wei Cai, Ling Ding, Juan Hu, Yong-xue Zhang, Guang-yao Huang, Pan Cheng, Shan Gao

**Affiliations:** Department of Pharmacology, Basic Medical College, Anhui Medical University, Hefei 230032, China

## Abstract

**Background:**

Xin-Ji-Er-Kang (XJEK) shows protective effects on the myocardial ischemic diseases in our previous reports. We hypothesized that XJEK may exert preventing effects on* L*-NAME induced hypertensive mice by ameliorating oxidative stress (OS) and endothelial dysfunction (ED).

**Methods:**

After treatment with XJEK for four weeks, cardiac function and cardiovascular pathology changes were evaluated. Then, endothelial-dependent vascular relaxation and serum NO, eNOS, AMDA, SOD, MDA content, and cardiac tissue eNOS expression were detected.

**Results:**

The hypertensive mice displayed distinct cardiovascular remodeling including increased HW/BW index (4.7 ± 0.33 versus 5.2 ± 0.34), cross-section area, and collagen deposition. In addition, ED was found manifested by decreased serum NO (20.54 ± 8.05 versus 6.29 ± 2.33), eNOS (28.34 ± 2.36 versus 20.37 ± 2.30), content, and decreased eNOS expression in cardiac tissue and damaged endothelium-dependent diastolic function. Moreover, OS was detected confirmed by decreased SOD activity and increased MDA content in serum. However, treatment with XJEK for 4 wk could reverse cardiovascular remodeling (HW/BW index normalized from 5.2 ± 0.34 to 4.59 ± 0.25), ameliorate and preserve endothelial function (NO: 16.67 ± 7.24 versus 6.29 ± 2.33; eNOS: 16.67 ± 7.24 versus 6.29 ± 2.33), and suppress OS.

**Conclusion:**

XJEK has protective effects against cardiovascular remodeling in L-NAME induced hypertensive mice.

## 1. Background

Cardiovascular diseases globally account for approximately 17 million deaths a year, specifically, one-third of premature deaths in man and one-quarter of premature deaths in women [1], among which hypertension complications give grounds for 9.4 million deaths throughout the world every year [1, 2]. No less than 970 million people all over the world are enduring hypertension and, even worse, they develop more cardiovascular complications [3, 4]. However, the causes of hypertension remain unknown in about 95% of all cases, despite the current assumption and extensive clinical and experimental research, and the main causes are usually attributed to genetic and environmental factors [5]. In essential hypertension, endothelial dysfunction is present in both resistance and conduit arteries, functioning as an early independent predictor of cardiovascular events [6, 7]. It has also been confirmed that nitric oxide generated by endothelial isoform of nitric oxide synthase (eNOS) in the vascular endothelium plays a crucial part in the regulation of blood pressure. Reduced nitric oxide bioavailability contributes to endothelial dysfunction and hypertension. The endothelial isoform of nitric oxide synthase (eNOS) is responsible for the production of nitric oxide within the endothelium. The loss of eNOS cofactor tetrahydrobiopterin to initial increase in oxidative stress leads to uncoupling of eNOS, in which the enzyme produces superoxide anion rather than nitric oxide, further substantiating oxidative stress to induce vascular pathogenesis. For the design of new options, it is crucial to have an in-depth understanding of the molecular mechanisms which regulate nitric oxide signaling under pathophysiological conditions [8].

A traditional Chinese herbal formula named Xin-Ji-Er-Kang (XJEK) is made of fourteen herbs such as* Panax ginseng *C.A. Mey.,* Polygonatum odoratum *(Mill.) Druce,* Astragalus mongholicus *Bunge,* Ophiopogon Japonicus *(Thunb.) Ker Gawl, and other ingredients. Both the basic research and clinical studies point to the protective role of XJEK in toxic myocarditis and viral myocarditis [9]. Our previous reports have confirmed that XJEK exerts protective effects on hypertension induced by 2-kidney 1-clip (2K1C) and ventricular remodeling in rats, the mechanism of which may be related to its antioxidant capacity [10].

This research aims to probe into the effects of XJEK on anticardiovascular remodeling in* L*-NAME induced hypertensive mice and explore its potential mechanisms especially underlying endothelial dysfunction (ED), in order to provide a scientific basis for the relevant clinical application.

## 2. Materials and Methods

### 2.1. Drugs and Reagents

The formula of XJEK was provided by Hefei Seven Star Medical Science and Technology Company (Hefei, China, as shown in Table 1), and the HPLC fingerprint and the results of XJEK extract preparation may refer to our previous reports [10]. N^*ω*^-nitro-*L*-arginine methyl ester (*L*-NAME) was purchased from Sigma-Aldrich (St. Louis, Mo, USA). Irbesartan was bought from Pfizer. Other chemicals in use were from commercial sources, conforming to reagent grade.

### 2.2. Animals and Experiment Protocols

Forty male Kunming mice (weight from 18 g to 22 g) were purchased from Shanghai Slac laboratory animal Corp. Ltd. [Certificate number SCXK (HU) 2012-0002]. On the basis of the Guide for the Care and Use of Laboratory Animals US National Institute of Health, a protocol can be outlined to guide relevant animal procedures. All animals were housed in specific pathogen-free (SPF) conditions (18~25°C, 50% humidity and alternating 12 h dark/night cycles). The performance has been approved by the committee on the Ethics of Animal Experiments of Anhui Medical University.

Forty mice were randomly divided into four groups as follows: (1) control group (*n* = 10), which were given tap water; (2)* L*-NAME group (*n* = 10), in which* L-*NAME (Sigma-Aldrich, St. Louis, Mo) was administered in drinking water at 2 mg/mL (approximately 160–170 mg/kg/day); (3)* L*-NAME + XJEK group, which received XJEK at 7.5 g/kg/day; (4)* L*-NAME + Irbesartan group, which received Irbesartan at 40 mg/kg/day. All animals were kept under standard conditions and fed with normal food and water ad libitum. Their body weight was monitored weekly. Treatment with XJEK and Irbesartan had been on for four weeks when hypertension was induced by the application of eNOS inhibitor* L*-NAME to drinking water, followed by an additional four weeks of continuous treatment. The animal experiment lasted for eight weeks, and then the final measurements and tissue harvest were performed.

### 2.3. Measurement of SBP

Prior to formal test, all mice adapted to breeding of one week. The mice were placed in the specialized holder for 10–15 minutes before the measurements to acclimate to their surroundings while preheated to dilate their tail vessels at 37°C; then tail SBP was measured in conscious mice (*n* = 10/group) at baseline and every week thereafter using a noninvasive tail-cuff device (ALC-NIBP, Shanghai Alcott Biotech Co. Ltd.) in a restrainer heated electrically to 37°C. The animals underwent at least three training sessions before initial baseline measurements, and SBP was determined as well.

### 2.4. Measurement of Hemodynamics

All subjects were anesthetized with pentobarbital sodium (45 mg/kg,* ip*) at the end of 8th week; then the neck was dissected, the right common carotid artery was isolated, and then a fiber-optic pressure recording system, Samba (Samba Sensor, Gothenburg, Sweden), had a thin fiber [outer diameter (OD) = 0.25 mm] and a pressure sensor (length and OD = 0.42 mm) attached to the end. For recording, the Samba sensor was implanted into the left ventricle along the right coronary artery; the signals could be noted on a BL-420S biological function experimental system (Chengdu Taimeng Software Co. Ltd., China). Heart rate (HR), left ventricular systolic pressure (LVSP), left ventricular end-diastolic pressure (LVEDP), maximal rate of pressure development for contraction (+*dp*/*dt*_max_), and maximal rate of pressure development for relaxation (−*dp*/*dt*_max_) were continuously recorded.

### 2.5. Cardiac Remodeling Index, Blood, and Tissue Sampling

Following the hemodynamic measurements, the left common carotid artery was isolated and then cannulated with a polyethylene catheter, and collected blood samples were placed into tubes containing anticoagulant at 3500 ×g for 10 min at 4°C. After centrifugation, the plasma was dispensed into sample tubes and stored at −80°C until being analyzed. Due to the requirement of heart and aorta collection, the still beating heart was exposed by opening the thoracic cavity after blood collection. The heart samples were divided into several parts to calculate the heart weight index (dividing heart weight by body weight, HW/BW) and various analyses; the aorta samples were applied for further study.

### 2.6. Experiments of Isolated Vascular Ring

A separate practice of the vascular ring experiments was performed, in accordance with the minor modification made in the previous description. The thoracic aorta was immediately excised and immersed in an ice-cold modified Krebs solution composed of the following (in mM/L): NaCl, 118; KCl, 4.75; NaHCO_3_, 25; MgSO_4_, 1.2; CaCl_2_, 2; KH_2_PO_4_, 1.2; glucose, 11. Transverse ring (approximately 4 mm in length) was cut and cleansed of all adherent connective tissues. A mixture of 95% O_2_/5% CO_2_ was gassed persistently, which was maintained at 37°C. A displacement transducer was created with rings whose resting tension stretched to 0.5 g; then it was inserted into the lumen and attached to the chamber to an isometric force. Each ring was equilibrated in an organ-bath solution for 1 h. Tissues were restretched and washed with warm Krebs solution every 15 mins during the experiment. With regard to the aorta ring's viability and endothelial integrity, they were confirmed with the concentration-relaxation response curves in response to acetylcholine (10^−8^–10^−4^ mol/L) and performed in undamaged rings precontracted by 10^−4^ mol/L phenylephrine. Vasorelaxation in response to acetylcholine was expressed as a percentage contraction, which was determined by the percentage of inhibition to the preconstriction tension.

### 2.7. Histological and Morphological Analyses of Heart and Thoracic Aorta

Paraffin-embedded heart tissue was cut into slides in 4 *μ*m and then stained with hematoxylin, eosin (H&E), and Van Gieson (V-G). With the software of NIH (National Institutes of Health Service Branch) Image 1.61 in digitalized microscopic images, the myocyte cross-sectional area (CSA), perivascular collagen area (PVCA), and collagen volume fraction (CVF) were analyzed.

Thoracic aorta tissues were fixed in 10% formalin solution, which were then paraffin-embedded and sectioned at 4 *μ*m thick through a rotary microtome and eventually underwent HE staining. The images were taken under microscopic observation; then total aorta (TAA), area of lumen (LA), CSA, aorta radius (AR), luminal radius (L), and media thickness (M) of aorta were determined using morphological image analysis system, and the ratio of M/L was calculated as previously described.

### 2.8. Measurement of Nitric Oxide (NO) and Endothelial NO Synthase (eNOS)

Due to the short half-life and low concentration of NO* in vivo*, we evaluated plasma NO levels by measuring its stable metabolites. However, as it is mostly unstable in physiological solution, NO was quickly converted to nitrite (NO_2_^−^) and nitrate (NO_3_^−^). The serum levels of NO_2_^−^/NO_3_^−^ were determined using NO detection Kit (Nanjing Jiancheng Bioengineering Institute, Nanjing, China) according to the manufacture's instructions. Briefly, nitrate was converted to nitrite with aspergillus nitrite reductase, and the total nitrite was measured with the Griess reagent. The absorbance was determined at 540 nm with a spectrophotometer. The content of eNOS was assessed by ELISA kit.

### 2.9. Determination of Malondialdehyde (MDA) Levels and Superoxide Dismutase (SOD) Activities

The instruction was suggested by the manufacturer (Nanjing Jiancheng Bioengineering Institute, Nanjing, China) and practiced as follows: to determine MDA content in plasma, thiobarbituric acid reactive substances (TBARS) were applied. The absorbance value at the wavelength of 532 nm could be measured, and nanomoles of MDA per milliliter could be expressed to identify MDA levels. Xanthine oxidase method was applied for the measurement of SOD activities. The absorbance value at 550 nm with the commercial kit could be determined, and relevant SOD activities could be expressed as units per milliliter.

### 2.10. Measurements of Asymmetrical Dimethylarginine (ADMA)

With reference to the instructions, this research determined the level of ADMA by using an ELISA kit that measured the optical density (OD) at 450 nm. By the comparison of OD of the samples to that of the standard curve, ADMA concentration in each sample was assessed after a series of treatment.

### 2.11. Immunohistochemistry of Endothelial Nitric Oxide Synthase

On the basis of instructions, immunohistochemical staining was performed by applying UltraSensitive S-P kit produced by Boster-Bio (China). In 10 mM sodium citrate (ph 6.0), sections were deparaffinized and microwave-treated for 10 mins twice. At a room temperature, the endogenous peroxidase was blocked in endogenous peroxidase blocking solution via 10 min of incubation. In a 1 : 100 dilution at 4°C, rabbit polyclonal antibodies against endothelial NO synthase (eNOS) were employed as primary antibodies for 18 h. Sections were washed for three times with phosphate-buffered saline (PBS) and thereafter incubated with biotin-conjugated anti-rabbit second antibody for 10 min. Another three times' washing with PBS was practiced before treatment of the sections with streptavidin-peroxidase for 10 min and then washed with PBS for three times again. Followed by haematoxylin counterstaining, ultimately, specimens were incubated in diaminobenzidine for 5 min. To record images, digital camera system (Leica DM IL, DC 300) was acquired throughout the entire sections.

### 2.12. Western Blotting

By using an extraction buffer, the total proteins were extracted from the cardiac tissues. To practice, the tissue was homogenized with RIPA lysis buffer containing PMSF on ice for 30 min and centrifuged at 12000*g* for 10 min at 4°C. By using bicinchoninic acid (BCA) method (BCA Protein Assay kit, Beyotime of Institute of Biotechnology, China), the protein in supernatants was determined spectrophotometrically at a 562 nm wavelength. 10% SDS-polyacrylamide gel electrophoresis (PAGE) was used to separate the total protein (100 mg) which was then transferred to a polyvinylidene difluoride (PVDF) membranes. The process lasted for 120 min at 100 mA. The membranes were then blocked with the buffer composed of 5% skim milk in TBS-T containing 10 mmol/L Tris-HCl (pH 6.8), 150 mmol/L NaCl, and 0.05% Tween 20 followed by an overnight incubation at 4°C with the primary antibodies of eNOS. 0.1% Tween-20 was applied for the washing of membranes for three times for 15 minutes and then the membranes were incubated subsequently with the appropriate secondary antibody for 1 h at 37°C. Super signal enhanced chemiluminescence (ECL) detection system was employed in the visualization of the blots according to the manufacturer's instructions, which then were calculated by Image J analysis software. GAPDH protein served as an internal calibration.

### 2.13. Statistical Analysis

Quantitative results are expressed as means ± S.D. Statistics were performed with one-way analysis of variance (ANOVA). Difference was taken statistically significant at *P* < 0.05.

## 3. Results

### 3.1. Effects of XJEK on SBP in* L*-NAME Induced Hypertensive Mice

**Figure 1:**
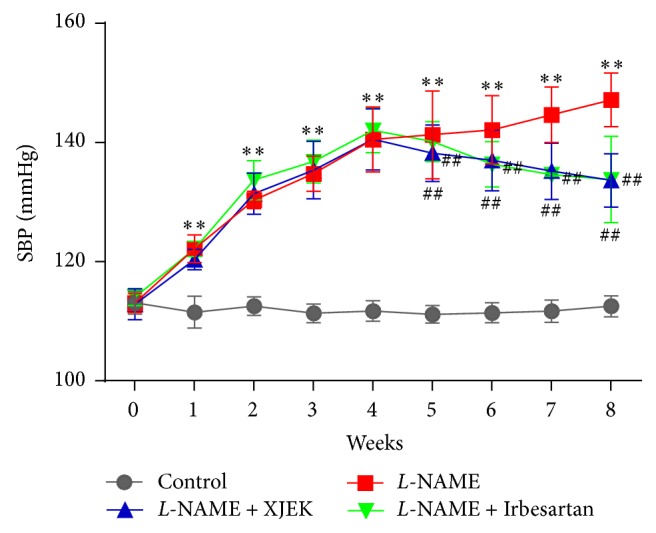
Effects of XJEK on SBP in* L-*NAME induced hypertensive mice during a 8 wk period. Nine time points of SBP were determined using tail-cuff apparatus measurement in each group. (mean ± SD, *n* = 8~10). ^*∗∗*^*P* < 0.01 vs. Control group; ^##^*P* < 0.01 vs.* L*-NAME group.

As shown in Figure 1, similar SBP was presented in different groups of mice before operation (*P* < 0.05). After treatment, SBP in the induced hypertension* L*-NAME group was markedly higher than that in control group (*P* < 0.01). SBP was considerably lower in hypertensive mice treated with XJEK and Irbesartan than that in* L*-NAME group from the 5th week (*P* < 0.01).

### 3.2. Effects of XJEK on Haemodynamic Parameters in* L*-NAME Induced Hypertensive Mice

The measurement of* in vivo *left ventricular function for all groups was carried out. As shown in Table 2, systolic cardiac parameters including LVSP, LVEDP, +*dp*/*dt*_max_, and diastolic cardiac parameter −*dp*/*dt*_max_ were all significantly elevated in* L*-NAME group compared to those in control group (*P* < 0.05 or *P* < 0.01). Nevertheless, treatment with XJEK and Irbesartan markedly inhibited the changes of hemodynamic parameters (*P* < 0.05 or *P* < 0.01).

### 3.3. Effects of XJEK on Cardiac Hypertrophy in* L*-NAME Induced Hypertensive Mice

**Figure 2:**
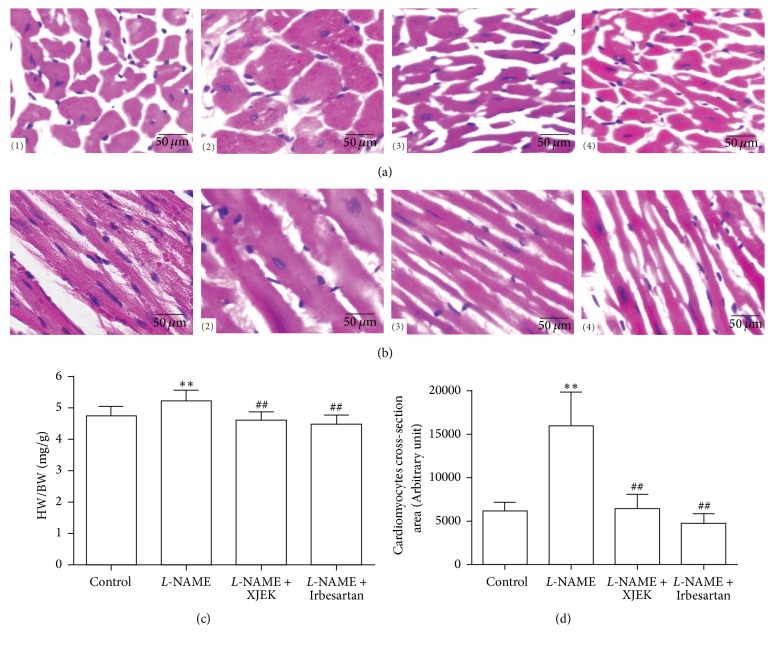
Effects of XJEK on HW/BW, cardiomyocyte CSA, and cardiomyocyte long axis in* L*-NAME induced hypertensive mice. (a) Representative images of histological section of cardiomyocyte long axis (HE staining, 400x); (b) representative images of histological section of cardiomyocyte long axis (HE staining, 400x); (c) ratio of heart to body weight (HW/BW, mg/g); (d) summary of CSA results analyzed by Image J. (mean ± SD, *n* = 8~10). (1) Control group; (2)* L*-NAME group; (3)* L*-NAME + XJEK 7.5 g/kg group; (4)* L*-NAME + Irbesartan 40 mg/kg group. ^*∗∗*^*P* < 0.01 versus control group; ^##^*P* < 0.01 versus* L*-NAME group.

As indicated by HE staining of cardiac tissues from* L*-NAME group, cardiac myocyte CSA and longitudinal diameter increased dramatically compared to control group (*P* < 0.01, Figures 2(a), 2(b), and 2(d)). HW/BW index was applied in the determination of the level of morphological hypertrophy of heart; the results illustrated in Figure 2(c) showed that an increase in HW/BW ratios was featured in comparison with control group (*P* < 0.01). XJEK and Irbesartan treatment could reverse those pathological changes for four weeks (*P* < 0.01).

### 3.4. Effects of XJEK on Cardiac Fibrosis in* L*-NAME Induced Hypertensive Mice

**Figure 3:**
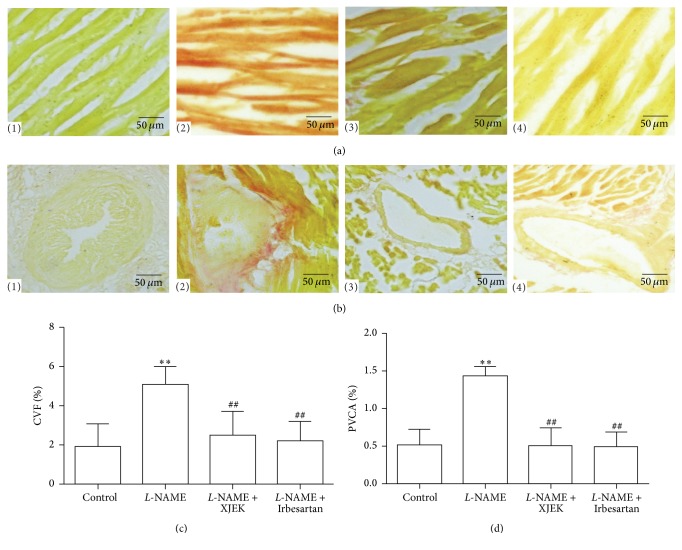
Effects of XJEK on cardiac fibrosis in* L*-NAME induced hypertensive mice. (a) Representative images of histological sections of myocardial fibrosis with VG staining (400x); (b) representative images of histological sections of perivascular fibrosis with VG staining (400x); (c) quantification results of myocardial fibrosis; (d) quantification results of perivascular fibrosis analyzed by Image J as % (mean ± SD, *n* = 8~10). (1) Control group; (2)* L*-NAME group; (3)* L*-NAME + XJEK 7.5 g/kg group; (4)* L*-NAME + Irbesartan 40 mg/kg group. ^*∗∗*^*P* < 0.01 versus control group; ^##^*P* < 0.01 versus* L*-NAME group.

The histological observation of the heart stained with VG staining in* L*-NAME induced mice revealed that the levels of collagen volume fraction (CVF) and perivascular collagen area (PVCA) significantly increased compared to those of control group (*P* < 0.01, Figure 3). Interestingly, treatment with XJEK and Irbesartan in mice significantly attenuated CVF and PVCA in the cardiac tissues (*P* < 0.01).

### 3.5. Effects of XJEK on Aortic Remodeling in* L*-NAME Induced Hypertensive Mice

**Figure 4:**
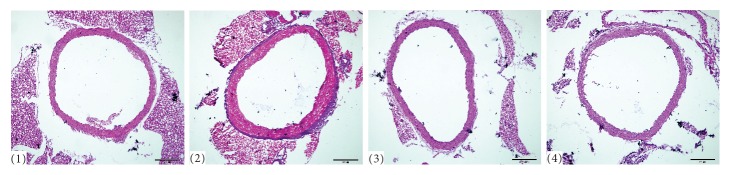
Representative figure of thoracic remodeling in different groups (HE staining, 100x). (1) Control group; (2)* L*-NAME group; (3)* L*-NAME + XJEK 7.5 g/kg group; (4)* L*-NAME + Irbesartan 40 mg/kg group.

The vascular remodeling of the thoracic aorta with exposure to* L*-NAME induced hypertensive mice was observed at the end of 4th week. Compared to control group, the values of the area of the TAA, LA, CSA, AR, M, and M/L ratio of the aorta in* L*-NAME induced mice were markedly upregulated (^*∗*^*P* < 0.05, ^*∗∗*^*P* < 0.01). These changes could be blocked by the continuing treatment of XJEK for weeks, and the positive drug Irbesartan exerted similar effects (*P* < 0.05 and *P* < 0.01, Table 3, Figure 4).

### 3.6. Effects of XJEK on Endothelial Dysfunction in* L*-NAME Induced Hypertensive Mice

**Figure 5:**
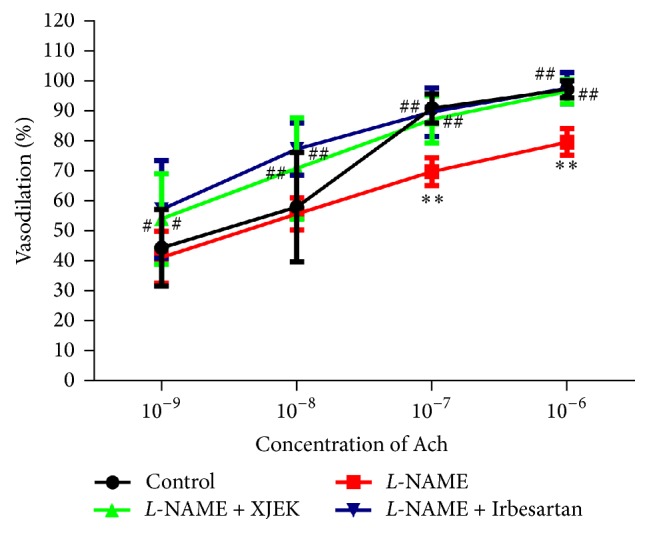
Effects of XJEK on aorta vasodilation in* L*-NAME induced hypertensive mice (mean ± SD, *n* = 8~10). ^*∗∗*^*P* < 0.01 versus control group; ^#^*P* < 0.05 and ^##^*P* < 0.01 versus* L*-NAME group.

Since reduced endothelial-dependent vasodilation was the main mechanism of* L*-NAME-induced hypertension, we investigated the dilative response to acetylcholine (Ach) in aortic rings stimulated by phenylephrine compared to those in control group (*P* < 0.01, Figure 5). The aortic rings obtained from* L*-NAME group with treatment of both XJEK and Irbesartan revealed an obvious increase in vasodilation induced by Ach compared to those from* L*-NAME group (*P* < 0.05, *P* < 0.01).

**Figure 6:**
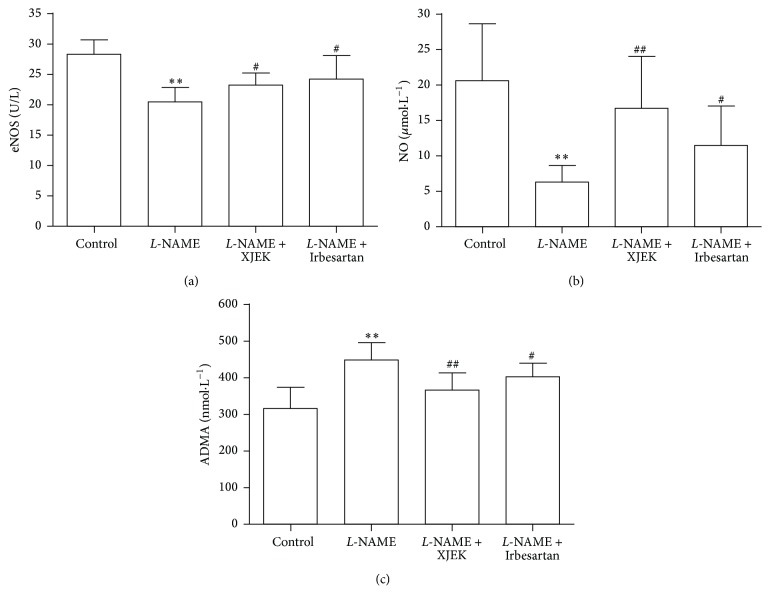
Treatment with XJEK alleviated endothelial dysfunction (mean ± SD, *n* = 8~10). (a) eNOS (U/L); (b) NO (*μ*mol/L); (c) ADMA (nmol/L). ^*∗∗*^*P* < 0.01 versus control group; ^#^*P* < 0.05 versus* L*-NAME group. ^##^*P* < 0.01 versus* L*-NAME group.

As expected, the contents of NO (6.29 ± 2.33 *μ*mol/L) and eNOS (20.37 ± 2.30 U/L) were markedly lowered in* L*-NAME group compared to control group (20.55 ± 8.05 *μ*mol/L, 28.34 ± 2.36 U/L). The reduced NO and eNOS levels were surprisingly elevated after XJEK and Irbesartan treatment. Remarkably higher NO and eNOS levels resulted from the performance of XJEK at (16.67 ± 7.24 *μ*mol/L, 23.22 ± 2.09 U/L), as well as that of Irbesartan (Figures 6(a) and 6(b)).

### 3.7. Effects of XJEK on Plasma ADMA in* L*-NAME Induced Hypertensive Mice

Our study showed that the level of asymmetrical dimethylarginine (ADMA) was significantly raised in* L*-NAME group compared with that in control group (*P* < 0.01), which, however, significantly decreased following XJEK and Irbesartan treatment. (Figure 6(c), *P* < 0.01 and *P* < 0.05).

### 3.8. Effects of XJEK on SOD Activities and MDA Levels in* L*-NAME Induced Hypertensive Mice

**Figure 7:**
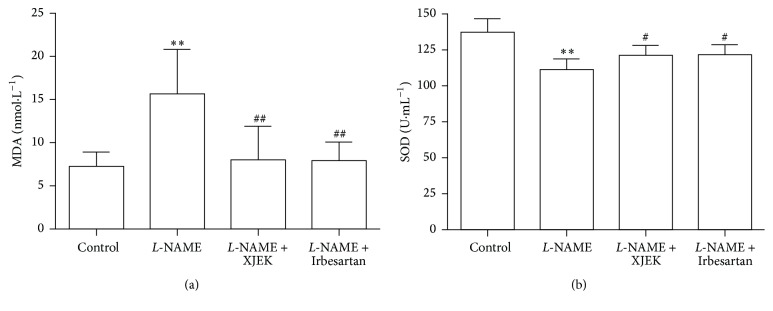
Effects of XJEK on SOD (a) and MDA (b) contents in* L*-NAME induced hypertensive mice.^*∗∗*^*P* < 0.01 versus control group; ^#^*P* < 0.05 and ^##^*P* < 0.01 versus* L*-NAME group.

Lower SOD was found in* L*-NAME group compared with that in control group, and XJEK as well as Irbesartan treatment restored SOD activity (Figure 7(b)). In contrast, MDA levels were boosted in* L*-NAME group on 4th week compared with those in control group. Treatment with XJEK and Irbesartan for four weeks markedly inhibited the increased plasma MDA (Figure 7(a)).

### 3.9. Nitric Oxide Pathway

**Figure 8:**
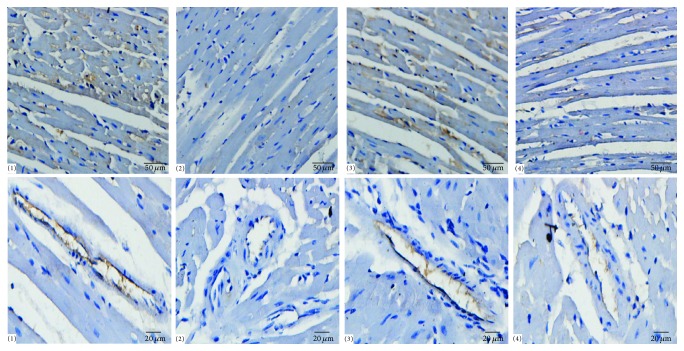
Endothelial nitric oxide synthase protein expression determined with immunohistological staining in interstitial tissues of myocardium and intramuscular arteries in hearts (200x, 400x) (mean ± SD, *n* = 8~10). Compared with control group, the eNOS protein expression levels in hearts of* L*-NAME group decreased significantly, which could be prevented by treatment with XJEK, just as the positive drug Irbesartan did.

Compared with mice of control group, the eNOS protein expression levels in hearts of* L*-NAME group decreased significantly (*P* < 0.01). XJEK obviously increased the levels of eNOS protein expression (*P* < 0.05), which could also be inhibited by treatment with Irbesartan (Figure 8). Thus, treatment with XJEK could enhance NO signaling system, which might contribute to reversing the cardiovascular remodeling.

### 3.10. Effects of XJEK on eNOS Expression in* L*-NAME Induced Hypertensive Mice

**Figure 9:**
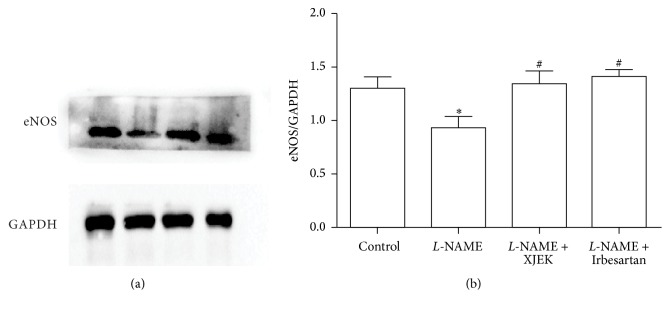
Effects of XJEK on eNOS expression (mean ± SD, *n* = 3). ^*∗*^*P* < 0.05 versus control group; ^#^*P* < 0.05 versus* L*-NAME group.

Figure 9 showed eNOS protein expression in hearts of* L*-NAME group animals (*P* < 0.05). Treatment with XJEK significantly increased the expression of eNOS, just as Irbesartan did (*P* < 0.05).

## 4. Discussion and Conclusions

The vascular endothelium is a single layer of dynamic cells which carries out important functions in the vascular homeostasis by means of multiple molecule synthesis and act as mediators of its autocrine and paracrine activity in response to different physical and chemical stimuli. Early work by Furchgott and Zawadzki showed that the presence of endothelium was vital to acetylcholine induced vasorelaxation in isolated artery preparations and this effect was attributed to a substance(s) released by the endothelium [11], which was subsequently identified as nitric oxide (NO) [12], initially named endothelium derived relaxing factor (EDRF) [13, 14]. Endothelial dysfunction (ED) resulted from an impairment of the endothelial NO activity, consequently leading to a reduction of the antithrombotic properties of the endothelium, vasomotor tone imbalance, increased permeability for plasmatic lipoproteins, enhanced cytokines and growth factors production, and circulating leukocyte hyperadhesiveness that is translated into an increased cardiovascular risk [15, 16]. Actually, it is crucial for vascular homeostasis regulation, an optimal relationship between NO, vasoconstrictor molecules produced by the endothelium, and the sympathetic nervous system. Therefore, the reduced NO bioavailability constitutes the early molecular basis of endothelial dysfunction and the biological link between cardiovascular risk factors and atherosclerosis [17].

One of the first diseases associated with reduced bioavailability of EDRF and altered vascular function is arterial hypertension. Hence, the experimental model of “N-nitro-L-arginine methyl ester (*L*-NAME)-induced” or “NO-deficient” hypertension [18] is established in our study to investigate the role of NO not only in vascular function and BP regulation but also in maintenance of homeostasis in the whole cardiovascular system. Due to chronic nonspecific inhibition of NO production, the development of ED is linked with a gradual elevation of BP, which exists as a dose-dependent model of hypertension and progressive vascular lesions [19, 20].

Cardiac hypertrophy is a common consequence of hypertension. Pathologic hypertrophy could contribute to the compromise of both diastolic and systolic function, which can be regarded as a result of volume or pressure overload or other hormonal or cytokine stimuli [21]. Administration with* L*-NAME results in the changes of cardiac hemodynamic parameters including LVSP, LVEDP, +*dp*/*dt*_max_, and −*dp*/*dt*_max_ which are sensitive indicators of cardiac function [22, 23]. The data from this study show that LVSP, LVEDP, +*dp*/*dt*_max_, and −*dp*/*dt*_max_ increase significantly in* L*-NAME group. The present study also reveals that* L*-NAME treatment leads to an elevation of the HW/BW ratio and CSA, an increase of collagen deposition, and an increase of SBP after only 1 week of* L*-NAME intervention and they present a continuous rising trend throughout the process of the study. In addition to the alteration in blood pressure, the changes can also be detected in vascular remodeling resulting from* L*-NAME by quantifying the extent of periaortic fibrosis in these animals, manifested as an elevation in wall thickness, TAA, and media thickness. Furthermore, the present results confirm that chronic oral therapy with XJEK as well as Irbesartan prevents hypertension and cardiovascular remodeling in* L*-NAME induced hypertensive mice [9].

Numerous studies show that the reactive oxygen species (ROS) level increases in the chronic hypertensive state, which is indicated by depletion of serum antioxidants and elevation of MDA level [24]. The increased blood pressure significantly produces ROS through vascular stimulation by mechanical stretch and activation of the renin-angiotensin system and further leads to oxidative stress [25, 26]. In oxidative conditions, NO produces peroxynitrite (ONOO^−^), a highly toxic and reactive radical, by the interaction with the superoxide anion [27]. The defense mechanism from the peroxynitrite radical is carried out by superoxide dismutase (SOD), which reduces the superoxide anion into hydrogen peroxide which subsequently is converted to water by the enzyme catalase. In states of high reactive oxygen species (ROS) generation (e.g., in presence of hyperglycemia, inflammatory states, and obesity smoke), the above antioxidant mechanisms are not sufficient. The subsequent increased oxidative status leads to the oxidation of numerous molecules, among which there are thiols, with consequent blunted NO bioavailability. At first, ROS can act as NO scavengers producing peroxynitrite. Moreover, the oxidation of the cNOS cofactor BH4 [28] induces cNOS uncoupling shifting to the production of superoxide instead of NO [29]. Lastly, ROS oxidizes the thiols with consequent reduced synthesis of RSNO and decreased activation of the sGC. Continuous oxidative situation can lead to the inability to restore normal reduced glutathione (GSH) or other thiol concentrations so that cGMP production becomes insufficient for a normal endothelial function.

Oxidative stress can also eventually account for the eNOS uncoupling by means of BH4 deficiency and asymmetric dimethyl-arginine (ADMA) increase. In particular, the high concentration of inflammatory cytokines (interleukin 6-IL-6 and TNFa) and the activation of the renin-angiotensin system induce ROS synthesis via NADPH oxidase (NOX) system, with consequent NO oxidation to peroxynitrite, resulting in reduced NO bioavailability. Peroxynitrite promotes the oxidation of the cofactor BH4 to dihydrobiopterin (BH_2_), inactivating it and therefore responsible for eNOS uncoupling. Moreover, the NOX system and peroxynitrite promote the oxidation of LDLs that reduce* L*-arginine bioavailability and, therefore, NO production. Ultimately, these conditions determine an increased production of ROS, a reduced NO synthesis, and augmented NO catabolism [30].

In our study, increased plasma ADMA concentrations have been detected in* L*-NAME induced hypertensive animal models, which has been widely shown to exert detrimental effects on vascular homeostasis by impairing endothelial function, increasing arterial stiffness, and promoting vascular inflammation [31, 32]. Asymmetrical dimethylarginine (ADMA), an endogenous methylated form of the amino acid* L*-arginine, inhibits the activity of the enzyme endothelial nitric oxide synthase (eNOS), with reduced synthesis of nitric oxide (NO) consequently [33]. Increased plasma ADMA can induce, in a sustained reduction in NO synthesis, renal perfusion and cardiac output, and a concomitant increase in blood pressure, peripheral vascular resistance, and sodium reabsorption in the kidney [34, 35], as well as increase arterial stiffness and reduce cerebral blood perfusion [36] in a dose-dependent fashion. The increased stiffness in arteries results in a higher systolic blood pressure [31]. Consequently, the rising cardiac afterload can boost not only the progress of left ventricular hypertrophy but also a prominent decrease in coronary perfusion pressure along with the onset of myocardial ischemia [36] and is significantly correlated with markers of abnormal left ventricular relaxation and diastolic dysfunction in patients diagnosed with chronic heart failure [37]. So ADMA can be a reliable biomarker of cardiovascular risk. In a large number of prospective clinical studies, ADMA has been characterized as a predictor of major cardiovascular events and mortality in patients with low, medium, and high cardiovascular risks [38, 39]. The present study demonstrates that treatment with XJEK significantly ameliorates oxidative stress via increasing SOD activities, and decreasing MDA formation and ADMA content.

Irbesartan, as a classic antihypertensive drug, falls to the category of angiotensin (Ang) п type 1 receptor (AT1R) blockers (ARBs). A survey of the available literature indicate that Irbesartan exerts antioxidative effects. Furthermore, Irbesartan may also block the generation of reactive oxygen species (ROS) [40, 41]. Irbesartan is selected in the present study as a positive drug since it satisfactorily illustrates the effectiveness of XJEK. Irbesartan administration markedly alleviates the increased HW/BW ratio and cardiac hypertrophy, reduces CVF and PVCA of heart and thoracic aorta, and improves histopathological injury. In addition, Irbesartan treatment markedly alleviates oxidative stress via promoting SOD activities and inhibiting MDA formation and ADMA content. These findings suggest that administration of Irbesartan has the potential to attenuate* L*-NAME induced hypertension.

Taken together, marked OS can be observed in* L*-NAME induced hypertensive mice, as is revealed by the present study, which participates in CR and ED, but the above effects are reversed by XJEK as well as Irbesartan treatment. It may be concluded that XJEK exerts protective effects on* L*-NAME induced hypertensive mice, and the pharmacological mechanism underlying may be attributed to the improvement of endothelial cell dysfunction, the decrease of ADMA content, and the attenuation of oxidative stress, at least in part.

## Figures and Tables

**Table 1:** Recipe of XJEK formulation.

Component	Voucher specimen number	Part used	Rate (%)
*Panax ginseng *C.A. Mey.	PCAHMU-20121005	Root	11.71
*Polygonatum odoratum* (Mill.) Druce	PCAHMU-20121006	Rhizome	7.03
*Panax pseudoginseng* var. Notoginseng (Burkill) G. Hoo & C.L. Tseng	PCAHMU-20121007	Root	3.09
*Allium macrostemon* Bunge	PCAHMU-20121008	Ramulus	7.80
*Angelica sinensis* (Oliv.) Diels	PCAHMU-20121009	Root	7.80
*Ophiopogon japonicus* (Thunb.) Ker Gawl.	PCAHMU-20121010	Root	7.80
*Schisandra chinensis* (Turcz.) Baill.	PCAHMU-20121011	Fruit	3.93
*Salvia miltiorrhiza* f. alba C.Y. Wu & H.W. Li	PCAHMU-20121012	Root	7.80
*Sophora flavescens* Aiton	PCAHMU-20121013	Root	7.80
*Glycyrrhiza acanthocarpa* (Lindl.) J.M. Black	PCAHMU-20121014	Rhizome	7.80
*Astragalus mongholicus* Bunge	PCAHMU-20121015	Root	11.69
*Epimedium acuminatum* Franch.	PCAHMU-20121016	Aerial part	7.80
*Trichosanthes obtusiloba* C.Y. Wu	PCAHMU-20121017	Seed	7.80
*Dryobalanops aromatica* C.F. Gaertn.	PCAHMU-20121018	Resin	0.15

**Table 2:** Effects of XJEK on cardiac function in *L*-NAME induced hypertensive mice (mean ± SD, *n* = 8~10).

Group	*n*	LVSP (mmHg)	LVEDP (mmHg)	+*dp*/*dt*_max_ (mmHg/s)	−*dp*/*dt*_max_ (mmHg/s)
Control	10	87.75 ± 14.85	−1.07 ± 6.69	4589.77 ± 844.2	−3861.16 ± 916.68
*L*-NAME	8	116.03 ± 14.83^*∗∗*^	−0.70 ± 5.62	5373.14 ± 854.29^*∗*^	−5115.99 ± 901.94^*∗*^
*L*-NAME + XJEK	9	108.58 ± 10.60	2.55 ± 4.87	4479.64 ± 858.25^#^	−3725.57 ± 949.41^#^
*L*-NAME + Irbesartan	9	91.36 ± 12.94^##^	−2.75 ± 5.47	4385.04 ± 899.64^#^	−3289.51 ± 959.93^##^

LVSP, left ventricular systolic pressure; LVEDP, left ventricular end-diastolic pressure; +*dp*/*dt*_max_, maximal rate of left ventricular systolic pressure; −*dp*/*dt*_max_, maximal rate of left ventricular diastolic pressure. ^*∗*^*P* < 0.05 and ^*∗∗*^*P* < 0.01 versus control group; ^#^*P* < 0.05 and ^##^*P* < 0.01 versus *L*-NAME group.

**Table 3:** Effects of XJEK on thoracic aorta remodeling in *L*-NAME induced hypertensive mice (mean ± SD, *n* = 8~10).

Group	*n*	TAA (×10^3^ *μ*m^2^)	LA (×10^3^ *μ*m^2^)	CSA (×10^3^ *μ*m^2^)	AR (*μ*m)	Lumen (*μ*m)	Media (*μ*m)	Media/lumen (%)
Control	10	464.93 ± 34.21	331.27 ± 31.10	133.66 ± 77.39	0.40 ± 0.02	0.34 ± 0.02	0.21 ± 0.01	0.64 ± 0.03
*L*-NAME	8	593.38 ± 43.90^*∗∗*^	390.13 ± 72.24^*∗*^	203.25 ± 28.67^*∗∗*^	0.45 ± 0.02	0.36 ± 0.03^*∗∗*^	0.26 ± 0.02^*∗∗*^	0.73 ± 0.11^*∗*^
*L*-NAME + XJEK	9	460.53 ± 58.00^##^	316.34 ± 58.86	144.19 ± 13.64^##^	0.40 ± 0.02	0.33 ± 0.03^##^	0.22 ± 0.01^##^	0.69 ± 0.08
*L*-NAME + Irbesartan	9	460.70 ± 80.48^#^	321.04 ± 67.77	139.66 ± 18.06^##^	0.39 ± 0.04	0.33 ± 0.04^#^	0.22 ± 0.01^##^	0.68 ± 0.13

TAA: total aorta area; LA: lumen area; CSA: cross section area; AR: aorta radius; LR: lumen radius; MT: media thickness. The vascular remodeling of the upper thoracic aorta observed at the end of the 4th week, which could be reversed by XJEK, so could the positive drug Irbesartan. ^*∗*^*P* < 0.05 and ^*∗∗*^*P* < 0.01 versus control group; ^#^*P* < 0.05 and ^##^*P* < 0.01 versus *L*-NAME group.

## Data Availability

The authors confirm that all data underlying the findings are fully available without restriction. All relevant data are within the paper and its Supporting Information files.
